# Rapid and Efficient Production of Coronary Artery Ligation and Myocardial Infarction in Mice Using Surgical Clips

**DOI:** 10.1371/journal.pone.0143221

**Published:** 2015-11-24

**Authors:** James N. B. M. de Andrade, Junnan Tang, Michael Taylor Hensley, Adam Vandergriff, Jhon Cores, Eric Henry, Tyler A. Allen, Thomas George Caranasos, Zegen Wang, Tianxia Zhang, Jinying Zhang, Ke Cheng

**Affiliations:** 1 Department of Molecular Biomedical Sciences and Center for Comparative Medicine and Translational Research, College of Veterinary Medicine, North Carolina State University, Raleigh, North Carolina, United States of America; 2 Joint Department of Biomedical Engineering, University of North Carolina at Chapel Hill and North Carolina State University, Raleigh, North Carolina, United States of America; 3 Department of Cardiology, The First Affiliated Hospital of Zhengzhou University, Zhengzhou, Henan, China; 4 Division of Cardiothoracic Surgery, University of North Carolina at Chapel Hill, Chapel Hill, North Carolina, United States of America; 5 The Cyrus Tang Hematology Center, Soochow University, Suzhou, Jiangsu, China; Georgia Regents University, UNITED STATES

## Abstract

**Aims:**

The coronary artery ligation model in rodents mimics human myocardial infarction (MI). Normally mechanical ventilation and prolonged anesthesia period are needed. Recently, a method has been developed to create MI by popping-out the heart (without ventilation) followed by immediate suture ligation. Mortality is high due to the time-consuming suture ligation process while the heart is exposed. We sought to improve this method and reduce mortality by rapid coronary ligation using a surgical clip instead of a suture.

**Methods and Results:**

Mice were randomized into 3 groups: clip MI (CMI), suture MI (SMI), or sham (SHAM). In all groups, heart was manually exposed without intubation through a small incision on the chest wall. Unlike the conventional SMI method, mice in the CMI group received a metal clip on left anterior descending artery (LAD), quickly dispensed by an AutoSuture Surgiclip^™^. The CMI method took only 1/3 of ligation time of the standard SMI method and improved post-MI survival rate. TTC staining and Masson’s trichrome staining revealed a similar degree of infarct size in the SMI and CMI groups. Echocardiograph confirmed that both SMI and CMI groups had a similar reduction of ejection fraction and fraction shortening over the time. Histological analysis showed that the numbers of CD68^+^ macrophages and apoptotic cells (TUNEL-positive) are indistinguishable between the two groups.

**Conclusion:**

This new method, taking only less than 3 minutes to complete, represents an efficient myocardial infarction model in rodents.

## Introduction

Coronary artery disease (CAD) is one of the major cardiovascular diseases that has caused serious effect on human health worldwide. It is a process of narrowing coronary arteries overtime involved at all stages of the atherosclerotic process, from lesion initiation to plaque rupture. Acute occlusion of the coronary artery by thrombosis could induce myocardial infarction (MI), and developed into heart failure eventually [1]. Both MI and heart failure remain major causes of mortality and morbidity.

Preclinical animal models are required to instruct how potential therapeutics such as stem cells [2] and nanoparticles [3] can modify the disease if they are to be developed for clinical uses. The wide range of genetically modified mice available makes this species an attractive model [4]. Although a variety of surgical manipulations have been used during the past decade to induce the ischemic event, permanent left anterior descending artery (LAD) occlusion is the most common model used by researchers[5, 6]. Nevertheless, this procedure is very time-consuming and associated with high surgery-related mortality, due at least in part to the trauma caused by mechanical ventilation and the large chest incision [7]. Recently, Gao et al developed an effective method to briefly expose the heart without mechanical ventilation [8]. This “heart popping-out” method has been identified as an effective way to induce MI [9, 10]. To avoid pneumothorax while the chest wall is open, it is essential to complete the procedure in a timely manner (e.g. < 1 min). LAD ligation using the suture is a rate-limiting step which normally takes 30 seconds to complete. We sought to develop a more rapid way for LAD ligation.

In this study, we used a quickly-dispensed surgical clip to ligate LAD and compared that to the classic suture ligation method. Surgical time, LAD ligation time, survival rate and degree of inflammatory response from both methods were analyzed and compared. Moreover, we evaluated the efficacy of this method by measuring cardiac function and infarct size.

## Materials and Methods

### 2.1 Ethics Statement

This study was carried out in strict accordance with the recommendations in the Guide for the Care and Use of Laboratory Animals of the National Institutes of Health. The protocol was approved by Institutional Animal Care and Usage Committee at North Carolina State University (Permit Number: IACUC# 13-128-B). All surgery was performed under isoflurane combined with oxygen, and all efforts were made to minimize suffering. For animal sacrifice, animals were euthanized through inhalation of CO_2_.

### 2.2 Experimental design

**Fig 1:**
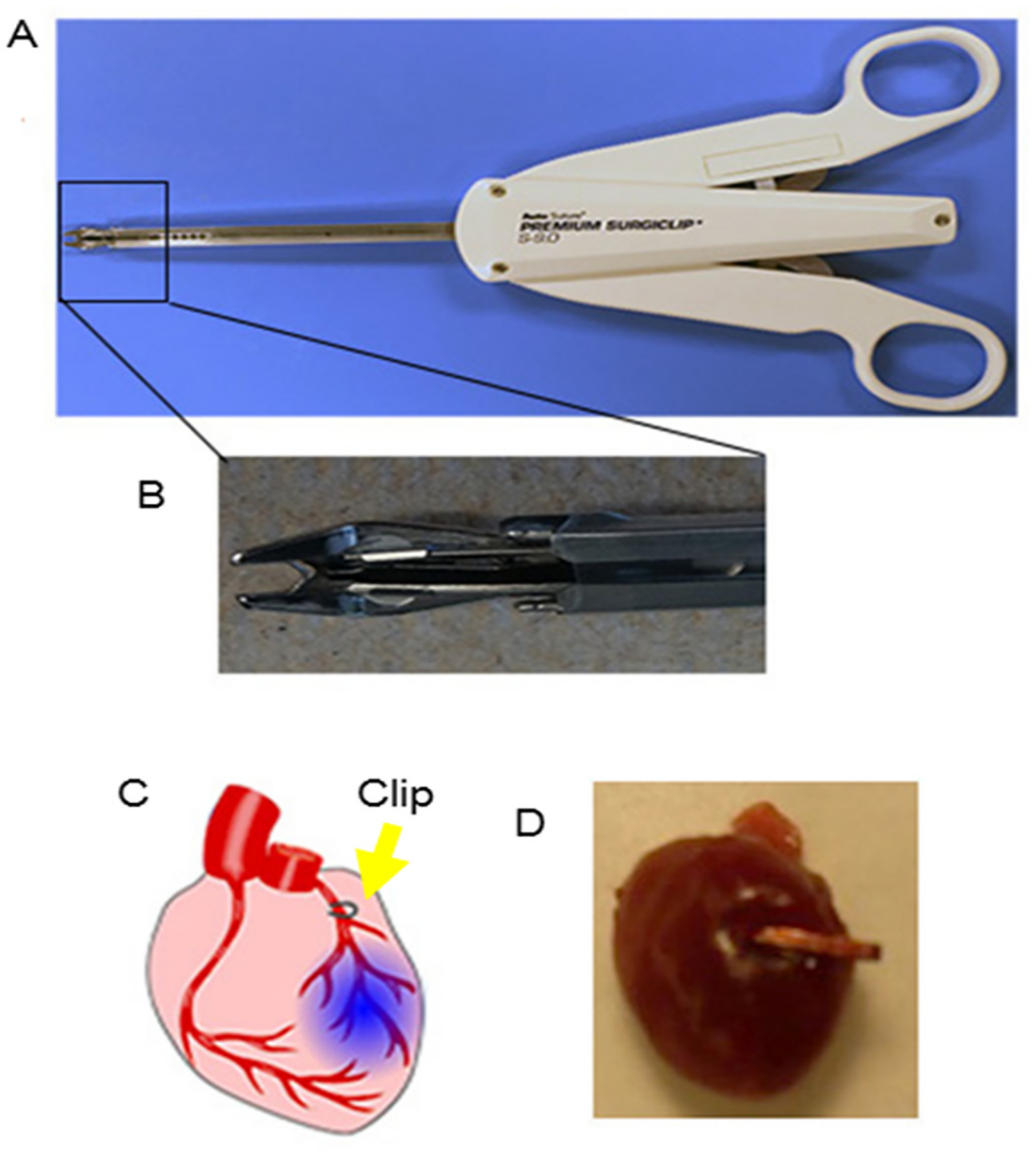
Surgical clip for LAD ligation. (A) & (B): Pictures showing the surgical clip dispenser and its tip containing the metal clip; (C) & (D): Schematic and gross heart picture showing left anterior descending artery (LAD) ligation by a surgical clip.

This study were conformed to the Guide for the Care and Use of Laboratory Animals published by the US National Institutes of Health. All animal work were compliant with Institutional Animal Care and Usage Committee at North Carolina State University (IACUC# 13-128-B). A total of 87 CD1 mice (male n = 18; female n = 69; outbred) were used for survival study. Additionally, 6 CD1 mice (male n = 2 per group; outbred) were used for 24 hour triphenyl tetrazolium chloride (TTC) staining. For the MI model, CD1 mice were subjected to permanent LAD ligation by either the metal clip method or classic suture method. CD1 mice were randomly assigned to three groups: suture MI (SMI) group, clip MI (CMI) group, and sham (SHAM) group. For animals in the SMI group, MI was induced by suture ligation of LAD. For animals in the CMI group, LAD ligation was performed using a quickly-dispensed metal clip using an AutoSuture Surgiclip^™^ (Fig 1A and 1B). Fig 1C and 1D show the positioning of the metal clip on the LAD. Total surgical time and time for LAD ligation were reported.

### 2.3 LAD ligation by the surgical clip method

**Fig 2:**
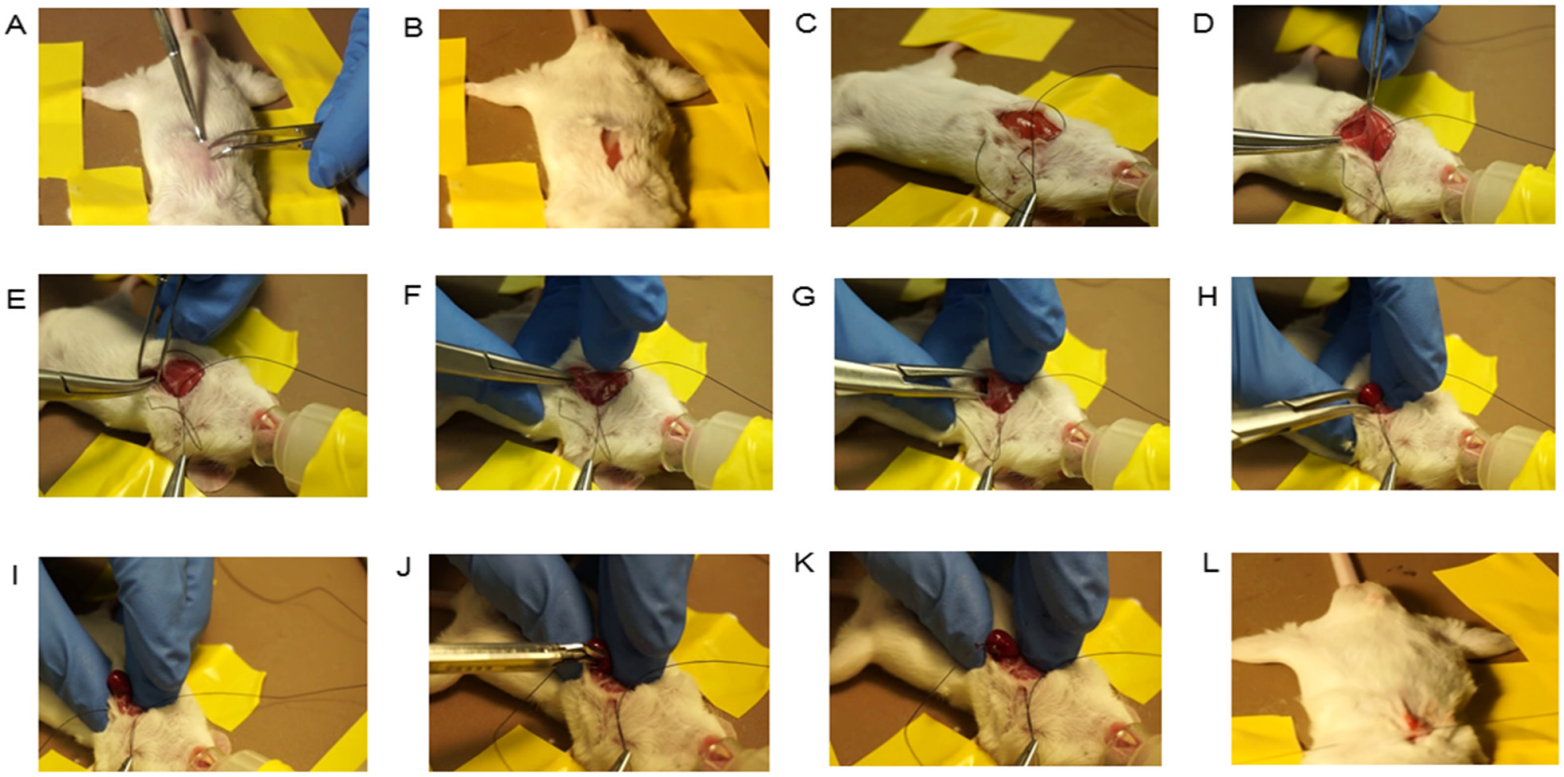
Various stages of clip method. (A): Placing the unconscious mouse on a warm pad and shaving in the area of the precordial chest; (B): A small skin cut was made in the middle position of precordial chest; (C): a purse suture was made; (D) Dissecting and retracting the pectoral major and minor muscle to expose the 4th intercostal space; (E): Placing the mosquito clamp at the 4th intercostal space with the help of forceps; (F): Forcing the mosquito to open the pleural membrane and pericardium at the 4th intercostal space; (G) & (H): With the clamp slightly open and finger strength, the heart was smoothly and gently “popped out” through the window; (I)–(K): Once the LAD was located, an automatic clip applier was used to dispense a clip to ligate the LAD with a ~60–90 degree angle along the heart long axis; (L): Placing the heart back in to the intra-thoracic space promptly after ligation, therewith manual evacuation of air and closure of muscle and the skin by the pre-placed purse-string suture.

MI induction was performed as described [8]. Briefly, mice were anesthetized with 3% isofluorane combined with 2% oxygen inhalation without mechanical ventilation. A small skin cut was made over the left chest and a purse suture was made by 4–0 silk suture (Fig 2A–2C). After dissection and retraction of the pectoral major and minor muscle, the 4th intercostal space was exposed (Fig 2D). A small window was made at the 4th intercostal space with a mosquito clamp to open the pleural membrane and pericardium (Fig 2E and 2F). With the clamp slightly open, the heart was smoothly and gently “popped out” through the window (Fig 2G and 2H). Once the LAD was located, an automatic clip applier (Premium Surgiclip S-9.0, Autosuture, USA) was used to dispense a small metal clip to ligate the LAD with a ~60–90 degree angle along the heart long axis (Fig 2I–2K). After ligation, the heart was immediately placed back into the intra-thoracic space followed by manual evacuation of air and closure of muscle and the skin by the pre-placed purse-string suture (Fig 2L). The mouse was then allowed to breathe room air and monitored during the recovery period. The mouse usually re-gained consciousness within 3 minutes. No artificial respiratory aid was required during the entire period. Movies depicting the entire procedure and alive mouse after surgery can be found in the online data supplement (S1 and S2 Videos).

### 2.4 LAD ligation by the standard suture method

Thoracotomy and heart exposition were performed as described in the section above. Once the heart was exposed, a 7–0 nylon suture was used to ligate LAD [5, 11, 12]. And we select the LAD position at a site about 2–3 mm from LAD origin [8,13]. The SHAM group underwent the same thoracotomy procedure except for the LAD ligation.

### 2.5 Heart morphometry analysis

For heart morphometry analysis, animals were euthanized through inhalation of CO_2_ at 21 days (after cardiac function assessment) and the hearts were harvested and frozen in OCT compound. Sections every 100 μm (5 μm thickness) were prepared. Masson's trichrome staining was performed as described by the manufacturer's instructions (HT15 Trichrome Staining (Masson) Kit; Sigma-Aldrich). Images were acquired with a PathScan Enabler IV slide scanner (Advanced Imaging Concepts, Princeton, NJ). From the Masson's trichrome-stained images, morphometric parameters including viable myocardium and scar size were measured in each section with NIH ImageJ software. The percentage of viable myocardium as a fraction of the scar area (infarcted size) was quantified as described [14].

### 2.6 Cardiac function assessment

Echocardiography was performed by a single observer, at 4h and 21 days after LAD occlusion, for the measurement of cardiac dimensions and function. Mice were anaesthetized with a 1.5% isofluorane-oxygen mixture and two-dimensional long axis images were record from the left caudal (apical) view, using a Philips CX30 ultrasound system couple with a L15 high-frequency probe. Two-dimensional guided M-mode images at chordae tendineae level were evaluated. M-mode measurements of left ventricle end-diastolic and end-systolic dimensions (LVEDD and LVESD, respectively) were performed by using the leading-edge method of the American Society of Echocardiograph [15]. For estimation of each parameter, the average of three measurements from three different cycles in an image was obtained. Left ventricular end-diastolic and systolic volumes (LVEDV and LVESV, respectively) were calculated by the biplane method of disks (modified Simpson’s rule). Ejection fraction (EF) was determined by using (LVEDV—LVESV/LVEDV) × 100%, and fractional shortening (FS) was calculated from the M-mode echocardiography images as (LVEDD—LVESD/LVEDD) × 100%.

### 2.7 Histology

For TTC staining, animals were euthanized through inhalation of CO_2_ then hearts were harvested 24 hours after MI and sectioned into five 1.2 mm-thick slices that were perpendicular to the long axis of the heart. The slices were then incubated in 1% freshly-made TTC solution at 37°C for 15 min and then digitally photographed. For Haematoxylin and Eosin (HE) staining, animals were euthanized through inhalation of CO_2_ at 21 days (after cardiac function assessment) and the hearts were harvested and frozen in OCT compound. Sections every 500 μm were prepared. Slides were fixed in Hematoxylin (Sigma-Aldrich, MO, USA) for 5 min at room temperature, and then rinsed in running water for 2 minutes; after decolorizing in acid alcohol for 2 seconds, rinsed again in sodium bicarbonate for 5 dips; rinsed out container with Dehydrant after 95% iso for 30 seconds, and then fixed in Eosin (Sigma-Aldrich, MO, USA) for 2 minutes, and then washed in dehydrant 100% (Richard-Allan Scientific, MI, USA) and Xylnene (VWR, PA, USA) for 3 times. The slides were digitally photographed. For immunofluorescence staining, mouse heart cryosections were fixed with 4% PFA, blocked/permeabilized with Protein Block Solution (DAKO) containing 1% saponin (Sigma-Aldrich, MO, USA), and then stained with anti-rat mononuclear phagocyte CD68 (1:200; BD, USA) antibodies. FITC or Texas-Red secondary antibodies were obtained from Abcam and used in conjunction with these primary antibodies. For TUNEL staining, heart cryosections were incubated with TUNEL solution (Roche Diagnostics GmbH, Mannheim, Germany) and counter-stained with DAPI (Life Technology, NY, USA). Images were taken by a Olympus epi-fluorescence microscopy system.

### 2.8 Survival Study

Totally 87 CD1 mice were used in this survival rate study (SMI, n = 37; CMI, n = 31; SHAM, n = 19). In order to reduce the suffering of mice, we set humane endpoints to decide when to sacrifice the mice. The humane endpoint included that body temperature and physical activity were significantly worse than the active mice and were decreased or not increase in a few hours, the animals did not response to intermittent stimulation 3 times in half one hour, or the respiratory rate of animals were rapidly or slowly apparently. Our animals used in this study were taken care by trained workers in College of Veterinary Medicine, North Carolina State University. They monitored the health of each animal every 6 h and strictly performed the rules of humane endpoints to determine when the animals should be euthanized by CO_2_. We analyzed the survival rate by statistics at two time points including 4 hour (perisurgical period) and 21 day (aftersurgical period) after surgery. After 21 day, all survived mice were euthanized by CO_2_ and some were harvested heart for histology study.

### 2.9 Statistical analysis

Results are presented as mean ± SD unless specified otherwise. Comparisons between any two groups were performed with two-tailed unpaired Student’s t-test. Comparisons among more than two groups were performed with one-way ANOVA followed by post hoc Bonferroni correction. For repeated measurements on time, the two-way ANOVA test was used. For the survival study, Kaplan-Meier analysis was used. Differences were considered statistically significant when *P*< 0.05.

## Results

### 3.1 The clip method reduces time required for surgery and LAD ligation

**Fig 3:**
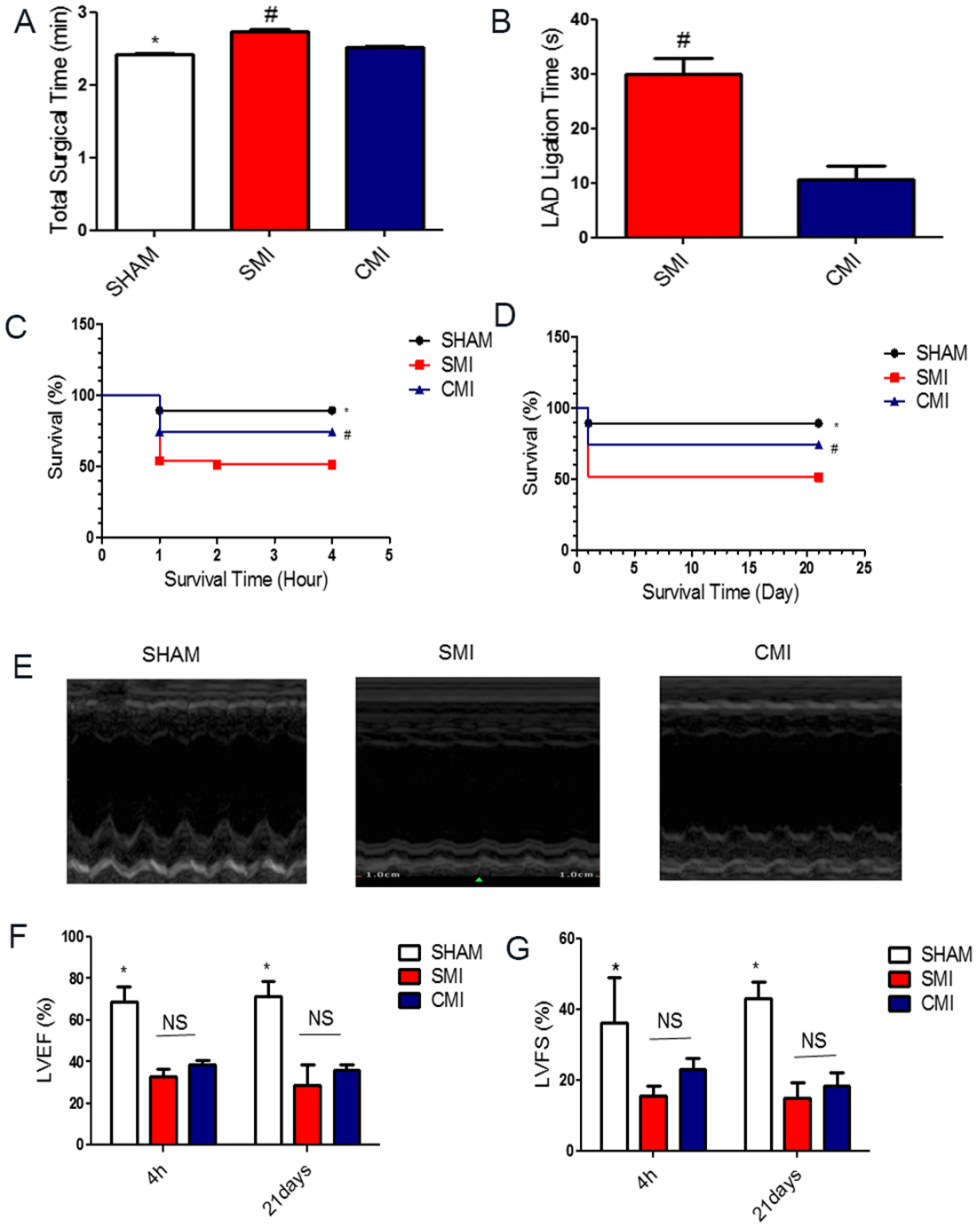
Total surgical time and LAD ligation time. (A): Total surgical time for the AMI model in SHAM (black bar), SMI (blue bar) and CMI (red bar) groups (n = 6); (B): Time required for left anterior descending artery (LAD) ligation in SMI (blue bar) and CMI (red bar) groups (n = 6). **Survival rates in the two MI models.** (C): Mice were subjected to SHAM or myocardial infarction (MI) surgery with either suture method or clip method and 4 hour perisurgery survival rate was recorded. (D): Long-term (21 day) survival rate. **Cardiac functions in the two MI models measured by echocardiography.** (E): Representative echocardiograph showing wall motion in SHAM, SMI and CMI groups at 21 day. (F & G): Left ventricular ejection fraction (LVEF) and left ventricular fraction shortening (LVFS) were measured by echocardiography at baseline (4 h post-MI) and 21 days afterward in SHAM, SMI and CMI groups (n = 6 animals per group). * indicates *P*<0.05 when compared to SHAM group; ^#^ indicates *P*<0.05 when compared to SMI group; NS indicates *P*>0.05 when compared to each other.

The total surgical procedure time was 2.73±0.41 min, 2.52±0.42 min, and 2.41±0.02 min for the SMI, CMI and SHAM group respectively (Fig 3A). While the total surgical time among the groups were not strikingly different, CMI drastically reduced the time needed for LAD ligation by ~3 fold (CMI: 10.50 ± 1.09 seconds vs. SMI: 30.00 ± 1.56 seconds) (Fig 3B). Without mechanical ventilation, this 20 seconds of reduction of heart exposure to room air can significantly improve the survival rate of the animals by reducing the risk of pneumothorax.

### 3.2 The clip MI method reduces mortality and improves survival

In total, we used 87 CD1 mice in this MI survival study. First, during the perisurgical period, which is the time from the beginning of the surgery until 4 hours after surgery, only 8 of 31 mice in the CMI group died, while 18 of 37 perisurgical deaths were seen in the SMI group (Fig 3C). Among the 8 died mice in CMI group, 4 died of bleeding, 3 died from pneumothorax and 1 died of sudden cardiac arrest. Among the 18 died mice In SMI group, 4 died of bleeding, 5 died from pneumothorax, 8 died of sudden cardiac arrest and 1 died of unknown reason. In the SHAM group, 2 of 19 mice died because of bleeding caused by the surgery. The overall survival rate 21 days after surgery was 89.5% in SHAM, 74.2% in CMI and 51.35% in SMI (Fig 3D). These data sets confirm that the clip MI method reduces mortality and improves survival.

### 3.3 The clip MI method is effective in inducing MI

Decrease of cardiac function is an ultimate indicator of a successful MI model (Fig 3E). Echocardiography was performed at 4 hours and 21 days after LAD ligation. Decreases in LV function were seen in mice in the SMI and CMI groups as compared to those in the SHAM group (Fig 3F and 3G). No significant differences in LVEF and LVFS were observed between the SMI and CMI groups at both 4 hours and 21 days post MI. Similarly, significant reduction in wall motion (Fig 3E) was observed in the SMI and CMI groups as compared to the SHAM groups at 21 day. Taken together, these results provide evidence for the effective induction of MI by the clip method.

### 3.4 Inflammatory response and cell death

**Fig 4:**
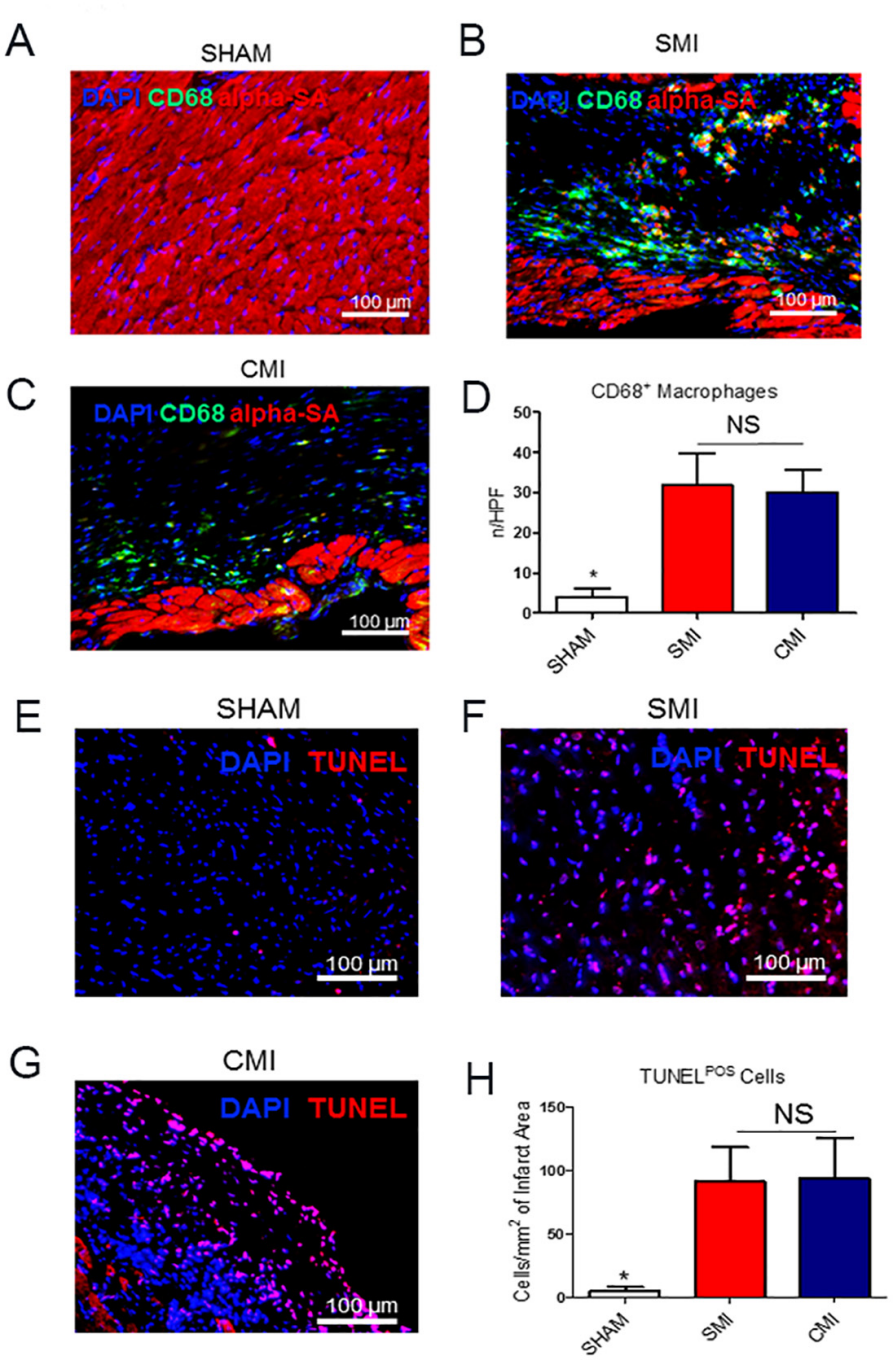
Inflammatory response in the two MI models. (A-C): Representative fluorescent micrographs showing the presence of CD68^+^ macrophages (green) in the hearts of SHAM (A), SMI (B), and CMI (C) groups. (D): Quantitation of CD68^+^ macrophages in each group at Day 21 (n = 3). **Cell apoptosis in the two MI models.** (E-G): Representative fluorescent micrographs showing the presence of TUNEL^+^ apoptotic cells (red) in the hearts of SHAM (E), SMI (F), and CMI (G) groups. (H): Quantitation of TUNEL^+^ cells (red) in each group at Day 21 (n = 3). Scale bars = 100 um. * indicates *P*<0.05 when compared to SHAM group; NS indicates *P*>0.05 when compared to each other.

To further determine the inflammatory responses to these two methods, the myocardial densities of CD68^+^ macrophages in the infarcted area were quantified 21 days after surgery. As expected, much less CD68^+^ macrophages were detected in the SHAM group (Fig 4A) than in the SMI (Fig 4B) or CMI (Fig 4C) group. The macrophage densities in the SMI and CMI groups are indistinguishable (Fig 4D), suggesting the metal clip did not exacerbate inflammation. Similarly, less TUNEL^+^ apoptotic cells were detected in the SHAM group (Fig 4E) than in the SMI (Fig 4F) or CMI (Fig 4G) group. However there is no difference between the SMI and CMI group (Fig 4H).

### 3.5 Infarct size

**Fig 5:**
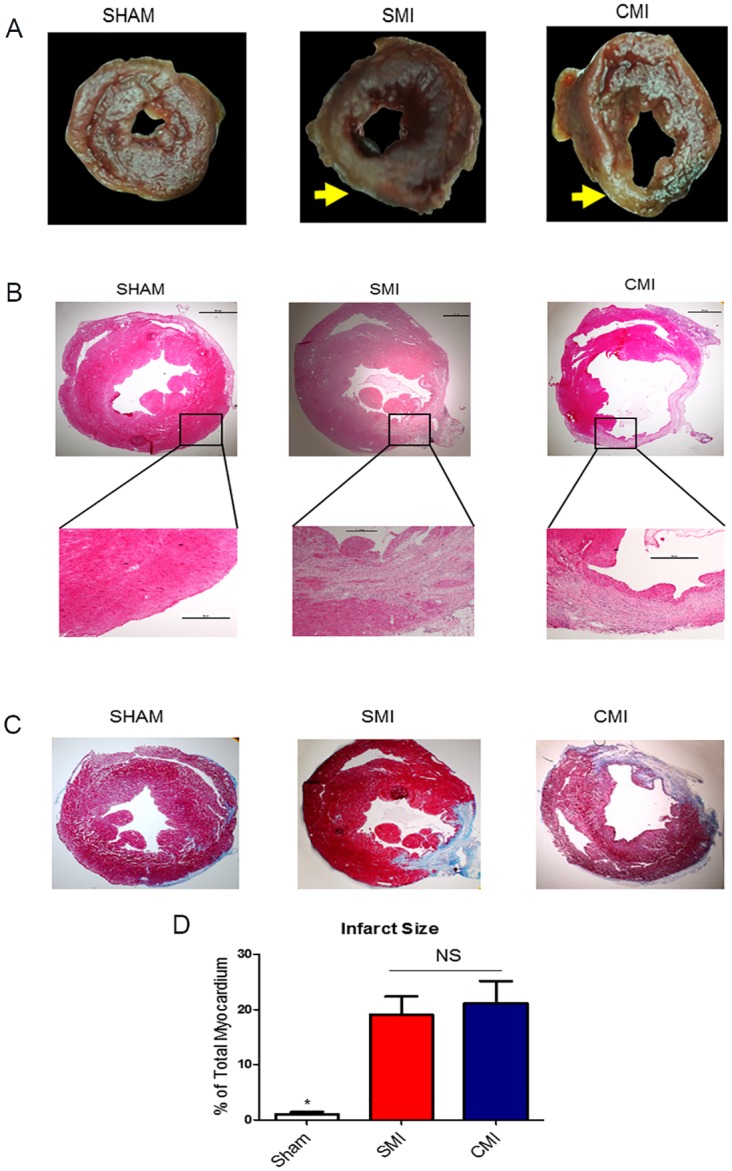
Short-term and long-term infarct sizes in the two MI models. (A): Representative photographs of TTC-stained heart sections obtained from SHAM, SMI and CMI groups at 24 hours after surgery, yellow arrow indicated infarcted area. (B) Representative micrographs of H & E stained heart sections obtained from SHAM, SMI and CMI groups at 21 day after surgery. (C) Representative micrographs of Masson’s trichrome stained heart sections obtained from SHAM, SMI and CMI groups at 21 day after surgery. (D) Quantitative analysis of infarct sizes from the Masson’s trichrome images (n = 6 animals per group). * indicates *P* < 0.05 when compared to SHAM; NS indicates *P*>0.05 when compared to each other.

TTC staining showed similar infarct sizes from the two methods 24 hours after surgery (Fig 5A). H & E staining 21 days after LAD ligation clearly identified the infarct in the CMI and SMI groups (Fig 5B). Snapshots of the infarct area showed no significant infiltration or foreign body reaction in both MI groups. Masson’s trichrome staining permits simultaneous detection of scar (blue) and healthy myocardial (red) tissues (Fig 5C). Quantitative morphometry at 21 days post-MI showed severe LV chamber dilatation and infarct wall thinning in the SMI and CMI groups as compared to SHAM group (Fig 5C). Infarct sizes were indistinguishable between the SMI and CMI groups (Fig 5D). These compound results suggest the new clip MI method is as good as the suture ligation method to produce a sizable infarct.

## Discussion

Coronary heart disease continues to be an epidemiologically and fiscally significant public health problem [13]. Myocardial ischemia triggers a complex sequence of pathophysiological responses involving inflammation, new tissue formation and post infarction tissue remodeling [16]. An acute myocardial infarction (AMI) model in rodents is commonly used to mimic human myocardial infarction as well as ventricular remodeling which can further lead to heart failure [4, 17].

Since the first mouse MI model was published in the year 1995, the permanent ligation of LAD has been considered as a standard model to cause myocardial injury for investigating pathophysiological mechanisms. Mechanical ventilation and a large chest incision are required to perform the procedure. A typical LAD ligation procedure can be very time-consuming, cause extensive tissue damage, high surgical-related death and can also be quite time consuming for most surgeons [18, 19, 20]. Recently, Gao et al reported an efficient method to expose the mouse heart and perform LAD ligation without, mechanical ventilation and a large incision on the chest wall [8]. However, as the time allowing for heart externalization should be no more than 30 seconds to limit global hypoxia, LAD ligation using sutures becomes a rate-limiting step [8].

In the present study, we developed a new method using a surgical clip instead of a suture to induce LAD ligation. Compared to the classic suture method, the clip MI method took only one third of the ligation time (Fig 3) and drastically increased survival rates (Fig 3). We also demonstrated that the clip MI method did not exacerbate myocardial inflammation (gauged by CD68^+^ macrophages, Fig 4) and tissue apoptosis (measure by TUNEL staining, Fig 4). Moreover, echocardiography confirmed that this new clip MI method had a similar reduction of ejection fraction and fraction shortening over the time (Fig 3) as compared to the suture method. To further evaluate the reliability of this new method of MI, we measured LV infarct size at 24 hrs by TTC staining and at 21 days by Masson’s Trichrome staining (Fig 5). No differences were found between the two methods. Moreover, the AMI model in rats has also been widely used to mimic human cardiovascular disease, studies are on-going in the lab to investigate whether the clip method can also be applied for creating myocardial infarction in rats. Clips with a larger size are available for such intervention in rats.

In sum, our study investigated surgical clip as a new method to induce myocardial infarction. This new method improves survival rate and reduces surgical time. Our findings provide impetus for employing this new myocardial infarction model in basic and translational researches for myocardial infarction.

## Supporting Information

S1 Video The entire procedure for inducing myocardial infarction by clip method. (AVI)

S2 Video After myocardial infarction surgery, the mice recovered from anesthetic. (AVI)
